# Cannabidiol for the treatment of refractory epilepsy in children: a critical review of the literature

**DOI:** 10.1590/1984-0462/2023/41/2021197

**Published:** 2022-07-06

**Authors:** Gabriela Araujo Moreira, Roddie Moraes, Ricardo Gullit Ribeiro, Ana Chrystina De Souza Crippa

**Affiliations:** a Universidade Federal do Paraná, Curitiba, PR, Brazil.

**Keywords:** Epilepsy, Cannabidiol, Drug resistant epilepsy, Child, Adolescent, Epilepsia, Canabidiol, Epilepsia resistente a medicamentos, Criança, Adolescente

## Abstract

**Objective:**

The aim of this current report was to present a critical review of the use of cannabidiol (CBD) in the treatment of refractory epilepsies in the pediatric population.

**Data source:**

Literature review was carried out in the Medline (PubMed), Cochrane, and Scientific Electronic Library Online (SciELO) databases with the descriptors “Cannabidiol” and “Epilepsy.” The search was not limited by the date of publication, language, or study design. A total of 69 articles were included in the review.

**Data synthesis:**

The efficacy of CBD in treating epileptic seizures has been confirmed by randomized controlled trials for Lennox–Gastaut syndrome, Dravet syndrome, and tuberous sclerosis complex. The incidence of side effects reported in subjects of the studies is high. However, most studies indicate a good safety profile and tolerance to the drug, with most of the adverse effects being mild to moderate and transient.

**Conclusions:**

There is no consensus on the release of CBD as a therapeutic tool by the drug regulatory agencies worldwide. However, the use of CBD is promising since it has presented satisfactory results in crisis control in well-designed studies. In addition, this drug has a good safety and tolerance profile. However, further studies with a long follow-up period are needed to confirm its usefulness and the long-term safety in pediatric patients.

## RESUMO

**Objetivo:**

Apresentar uma revisão crítica do uso de canabidiol no tratamento de epilepsias refratárias na população pediátrica.

**Fontes de dados:**

Revisão da literatura realizada nas bases de dados Medical Literature Analysis and Retrieval System Online (Medline/PubMed), Cochrane e Scientific Electronic Library Online (SciELO), com os descritores “*cannabidiol*” e “*epilepsy*”. A busca não foi limitada por data de publicação, idioma ou desenho do estudo. Sessenta e nove artigos foram incluídos na revisão.

**Síntese dos dados:**

A eficácia do canabidiol no tratamento de crises epilépticas foi confirmada por ensaios clínicos randomizados para síndrome de Lennox-Gastaut, síndrome de Dravet e complexo de esclerose tuberosa. A incidência de efeitos colaterais relatados nos indivíduos dos estudos é alta. No entanto, a maioria dos estudos indica um bom perfil de segurança e tolerância ao medicamento, com efeitos adversos leves a moderados e transitórios.

**Conclusões:**

Não há consenso sobre a liberação do canabidiol como ferramenta terapêutica nas diversas agências reguladoras de medicamentos em todo o mundo. Entretanto, seu uso é promissor, uma vez que apresentou resultados satisfatórios no controle de crises em estudos bem delineados. Além disso, esse medicamento apresenta um bom perfil de segurança e tolerância. No entanto, mais estudos com longo período de acompanhamento são necessários para confirmar sua utilidade e sua segurança em longo prazo em pacientes pediátricos.

## INTRODUCTION

Epilepsy is the most common chronic neurological condition during the first 10 years of life. One in five children with epilepsy may experience repeated cycles of relapse and remission or may be affected by a form of epilepsy refractory to conventional treatment. Patients with these forms of severe refractory epilepsies are at increased risk of accidents, unexpected sudden death, and respiratory infections.^1^ Thus, the development of new drugs to treat this condition is of fundamental importance.

Interest in cannabidiol (CBD) for the treatment of refractory epilepsy in the pediatric population has grown in the past decade. CBD is a *Cannabis sativa-*derived compound that has emerged as an attractive candidate due to its comparatively favorable therapeutic index and the probable lower incidence of undesirable psychoactive properties.^2^

In recent years, European, U.S., and Brazilian Pharmacological companies have actively engaged in developing the formulations of highly purified liquid CBD (CBD solution in sesame, corn, and other oils) as a potential anticonvulsant medication. The sesame oil formulation (Epidiolex®) was approved by the U.S. Food and Drug Administration (FDA) in 2018, for the treatment of seizures associated with the Dravet syndrome (DS) and Lennox-Gastaut syndrome (LGS) in individuals aged 2 years and older.^3^ Later, Brazilian researchers studied the specific use of CBD to treat refractory epilepsies in children.^4^

The endocannabinoid system (ECS) is composed of cannabinoid receptors (CB), endogenous fatty acid ligands, and proteins responsible for the biosynthesis and degradation of endocannabinoids which has multiple functions under physiological and pathological conditions.^5^ The cannabinoid receptors, that is, CB1 and CB2, are coupled to G protein and are found in the central nervous system (CNS), some peripheral nerves, and many blood cells.^5,6^ Both CB1 and CB2 receptors can be activated by endocannabinoids but exhibit different affinities and biological effects.^6^ CB1 receptors are found in large numbers in the neocortex, basal nuclei, hippocampus, amygdala, and interneurons of the dorsal horn of the spinal cord.^5–7^ CB1 receptors in the hippocampus are mainly located in presynaptic GABAergic neurons containing basket cells.^7^ CB2 receptors in the CNS are mainly found in microglia, although there is evidence in neurons of the afferent pain pathways.^6^

Some reports^6–9^ indicated that individuals with refractory epilepsy could benefit from the use of CBD. The studies varied from cases in which seizures were reduced to others that reported complete cessation of seizures and/or mixed findings.^6–8,10^ In several animal models of generalized and focal-onset epilepsy, CBD has been shown to have anticonvulsant properties capable of improving neuronal hyperexcitation related to symptoms.^4,5^ Some authors^4,5^ also proposed that endocannabinoids regulate the duration and end of seizures, activating the CB1 receptor and making CBD an attractive anticonvulsant potential in the pediatric population.

Genetic variability influenced the action of CBD, being one of the confounding factors in the interpretation of study data.^6^ Because of this fact, many authors have studied the mechanisms responsible for CBD’s anticonvulsant therapeutic benefits.^4–6,8,9^ CB receptors are presynaptically expressed in GABAergic glutamatergic and interneuron and can thus modulate the release of neurotransmitters.^6^ Most conventional antiepileptic drugs work to reduce neuronal excitability by blocking the release of excitatory neurotransmitters, such as glutamate (Glu), or by increasing the release of inhibitory neurotransmitters, such as GABA.^11^

Neurophysiological research has confirmed that glutamic acid is the primary stimulant of the CNS. Increased Glu release causes toxicity and seizures due to intense excitability in rodents and primates.^5^ The main result of the activation of the CB1 receptor is the reduction of the presynaptic release of Glu.^5^ Therefore, CBD could provide neuroprotection against acute excitotoxicity by indirect activation of CB1 since it is a negative partial allosteric modulator of CB1 and CB2, whose anticonvulsant effect is independent of the activation of the ECS.^4,5,8^ During an epileptic seizure, excess Glu release from presynaptic excitatory neurons results in hyperactivation of presynaptic CB1 receptors. The negative feedback mechanism provided by CB1 receptors decreases the release of more Glu and prevents an intensification of neuronal hyperexcitability, which can lead to the cessation of seizures.^4^

Although CBD has a low affinity for CB1 and CB2 receptors, it can be used to indirectly modulate these receptors and improve the actions of the ECS through various mechanisms.^6^ The first of the mechanisms is to prolong the effect of anandamide by inhibiting its uptake.^6^ This result has shown mixed effects on seizures induced by pentylenetetrazole — a CB1-dependent seizure drug used in animal models of seizures.^12^ The anticonvulsant effect was observed^12^ at lower doses of the inhibitors, and a pro-convulsive effect was observed at higher doses. This observation suggests that the extracellular accumulation of anandamide has anticonvulsant effects through the CB1 receptor, while the intracellular accumulation of anandamide may be pro-convulsive. Moreover, CBD inhibits 5-lipoxygenase and amide hydrolase from fatty acids, both enzymes being responsible for the degradation of endocannabinoids.^6^

The evidence is that antiepileptic drugs also target voltage-dependent ion channels, such as sodium and calcium channels, where some drugs act in one of the two channels and others both.^5,6^ CBD is an allosteric modulator antagonist of the GPR55 receptor in the CNS, repressing the release of intracellular calcium and controlling the neuronal hyperactivation characteristic of epilepsy.^12^ Interestingly, CBD was demonstrated to desensitize the potential of vanilloid receptors, i.e., TRPV1 and TRPV2, preventing the secretion of extracellular calcium ions and negative regulation of neuronal hyperexcitability and pointing to another potential anticonvulsant mechanism. The authors also reported that CBD partially improves microglial phagocytosis in rodents by activating TRPV1 and probably TRPV2.^5,12^ Accordingly, in a retrograde way, these endocannabinoids bind to presynaptic CB1 receptors and decrease the release of neurotransmitters, resulting in the blocking of calcium channels and stimulating the potassium channels to inhibit the release of neurotransmitters.^11^

Another potential route of anticonvulsant activity for CBD could be the inhibition of the nucleoside balancing transporter 1 (ENT1) involved in the synaptic uptake of adenosine, thus increasing extracellular adenosine. Increased levels of extracellular adenosine, in turn, decrease neuronal hyperexcitability and neurotransmission.^13^

The present review provides an overview of the literature evidence of the physiological properties of CBD, doses, routes of administration, indications, and regulatory agencies for the treatment of refractory epilepsies in the pediatric population.

## METHOD

The aim of this integrative review was to describe the state of the art of CBD use in the pediatric population based on the question “When is cannabidiol indicated in childhood epilepsy?” Studies that met the following inclusion criteria were considered eligible: observational studies, interventional studies, systematic reviews, and meta-analyses, in the past 10 years, in the English language. Studies carried out exclusively with adults were excluded.

The review was carried out on Medline (PubMed), Cochrane, and Scientific Electronic Library Online (SciELO) databases between January and April 2021, including articles published between 2011 and 2021 in Portuguese and English languages. The descriptors to be used were defined in the Health Sciences Descriptors (DeCS) and the Medical Subject Headings (MeSH): “Cannabidiol” AND “Epilepsy.” In addition, to minimize possible losses of publications, a manual search from the list of references of the articles included in the review was performed to detect other articles not included by the search strategy in the cited databases.

**Figure 1:**
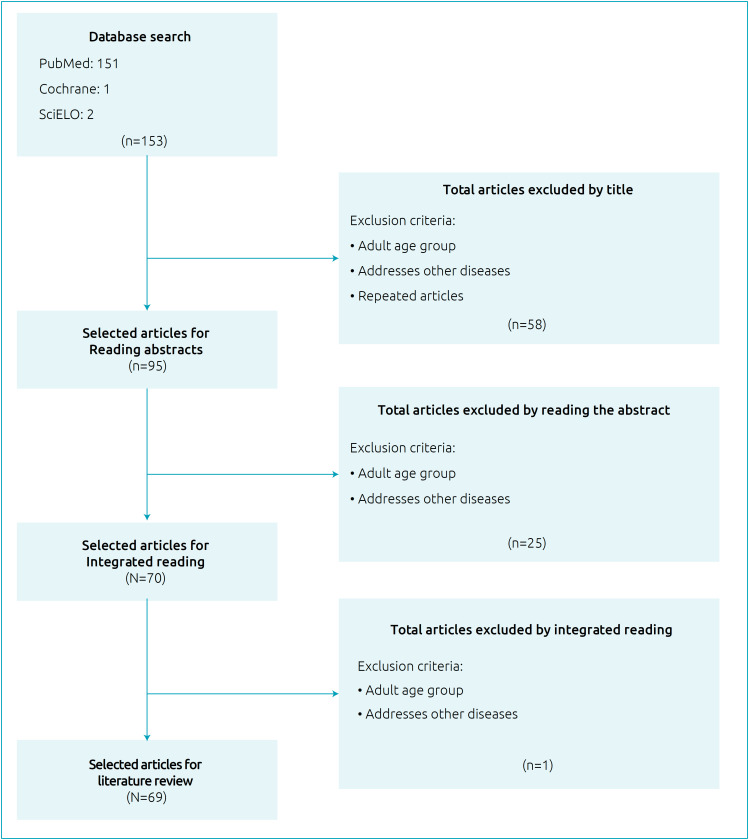
Flowchart of study retrieval and selection.

Initially, the titles of the articles were read by two independent researchers, and the inappropriate ones were excluded. Then, the abstracts were read, and the suitable articles were selected for reading in full. Subsequently, articles not relevant to the topic were excluded, and an integrative review was executed. For all stages, articles that addressed the use of CBD in the pediatric population were used as inclusion criteria, and studies conducted exclusively with adults and those who addressed the use of CBD in diseases other than epilepsy were used as exclusion criteria. The differences between the researchers during the processes were resolved by consulting a third researcher. The study selection flowchart is shown in Figure 1.

## RESULTS AND DISCUSSION

The effectiveness of CBD in the treatment of epileptic syndromes has already been confirmed in well-designed clinical trials for LGS, DS, and tuberous sclerosis complex. Current evidence in the literature suggests that, even with a high rate of adverse effects in the studies, CBD is a safe drug, since most side effects were classified as mild or moderate and transient.

Currently, there is no consensus regarding the release or prohibition of CBD for the treatment of diseases; there is still a lot of mystification and misinterpretation about cannabis, cannabinoids, and CBD. In the United States, the FDA in June 2018 released the purified CBD formulation for the treatment of seizures associated with LGS or DS in patients aged 2 years or older, based on studies that are detailed below.^14^ In the European Union in 2019, the European Medicines Agency (EMA) also released the purified CBD as a treatment for DS and LGS, however, in conjunction with clobazam.^14^ In 2020, the FDA also approved the use of tuberous sclerosis (TSC).^15^ In contrast, in Brazil, ANVISA (National Health Surveillance Agency) does not supply the CBD, as it only authorizes its import. The authorization is valid for 2 years and requires a medical prescription and specification of the quantity to be imported.^16^

Devinsky et al.^17^ described the first prospective, open-label, multicenter intervention study in 2016 that recruited and analyzed the efficacy of CBD in 137 patients who aged 1–30 years (median age: 10.5 years) with childhood-onset treatment-resistant epilepsy (45% was DS or LGS). The methodology was to add oral CBD to current therapy with an initial dose of 2–5mg/kg/day until intolerance or 25–50mg/kg/day over a 12-week treatment period. Considering all types of seizures, 51 (37%) patients had a reduction of 50% or more, 30 (22%) patients had a response of 70% or more, and 11 (8%) patients had a response of 90% or more. Patients (n=32) with DS were recorded a median reduction of 42.7% in total monthly seizures of all seizure types, and 4 (13%) were free of all seizure types. Patients (n=32) with LGS were recorded a median reduction of 35.5% in total monthly seizures of all types, but no one was seizure-free. From the data of this open intervention study, randomized clinical studies were carried out to investigate the effect of CBD on DS and LGS.

In 2017, Devinsky et al.^18^ described a double-blind, placebo-controlled, multicenter trial with 120 patients who aged 2–18 years with DS and drug-resistant seizures, randomized them to receive oral CBD (20mg/kg/day) or placebo in addition to standard antiepileptic treatment, and then compared the change in convulsive-seizure frequency over a 14-week treatment period between the groups. The adjusted median difference between the CBD group and placebo in the change in seizure frequency was -22.8%, and the percentage of patients who became seizure-free was 5% with CBD and 0% with placebo.

For LGS, two randomized, double-blind, placebo-controlled, multicenter trials were conducted. First, Thiele et al.^19^ performed a trial in 171 patients (age 2–55 years, with a median age of 14.2) with LGS who did not respond to treatment with at least two antiepileptic drugs. The patients were randomized to receive CBD (20mg/kg/day) or placebo in addition to standard therapy over 14 weeks. The median percentage reduction difference in monthly drop-seizure frequency from baseline between the groups was -17.21%. In contrast, Devinsky et al.^20^ enrolled 225 patients with LGS (age range 2–55 years) who had at least two types of generalized seizures for at least 6 months and randomized them to receive CBD 20mg/kg/day or CBD 10mg/kg/day or placebo over 14 weeks. The median percentage reduction from baseline in drop-seizure frequency during the treatment period was 41.9% in CBD 20mg/kg/day, 37.2% in CBD 10mg/kg/day, and 17.2% in placebo group.

Thiele et al.^21^ performed a double-blind, placebo-controlled, multicenter trial to evaluate the efficacy of CBD as a complementary treatment to TSC with medication-resistant epilepsy. The sample comprises 224 patients aged 1–65 years, although most of them (n=166) aged between 1 and 17 years. They were randomized to receive CBD 50mg/kg/day or CBD 25mg/kg/day or placebo for 16 weeks. The percentage reduction in total seizures was 29.1% between placebo and CBD 25mg/kg/day and 28.4% between CBD 50mg/kg/day and placebo.

All the studies discussed, with their study design, sample, main results and dose of CBD, are summarized in Table 1.

**Table 1:** Human studies on the effectiveness of cannabidiol.

Study design	Sample	Results	Dose	Reference
Prospective, open-label, multicenter, interventional trial	137 patients (age range, 1–30 years) with childhood-onset treatment-resistant epilepsy	All types of seizures: 37.0% had a reduction of 50.0% or more. DS (n=32): median reduction of 42.7% in monthly total seizures for all seizure types and 13.0% were free of all seizure types LGS (n=32): median reduction of 35.5% in monthly total seizures for all types.	CBD 25–50mg/kg/day for 12 weeks	Devinsky et al.^17^
Prospective, double-blind, placebo-controlled, multicenter trial	120 patients (age range, 2–18 years) with DS and drug-resistant seizures	Adjusted median difference between CBD and placebo: seizure frequency was − 22.8 percentage points (p=0.010). Percentage of patients who became seizure-free: 5.0% with CBD vs. 0.0% with placebo (p=0.080).	CBD 20mg/kg/day for 14 weeks	Devinsky et al.^18^
Prospective, double-blind, placebo-controlled, multicenter trial	171 patients (age range, 2–55 years) with LGS that not responded to treatment	Median percent reduction in drop-seizure frequency from baseline: −17.2 (p=0.014) with CBD vs. placebo.	CBD 20mg/kg/day for 14 weeks	Thiele et al.^19^
Prospective, double-blind, placebo-controlled, multicenter trial	225 patients (age range, 2–55 years) with LGS	Median percent reduction in drop-seizure frequency: 41.9% in CBD 20mg/kg/day, 37.2% in CBD 10mg/kg/day, and 17.2% in placebo group (p<0.050)	CBD 10 or 20mg/kg/day for 14 weeks	Devinsky et al.^20^
Prospective, double-blind, placebo-controlled, multicenter trial	224 patients (age range, 1–65 years; 166 between 1 and 17) with TSC medication-resistant epilepsy	Percentage reduction in total seizures: 29.1% (p=0.001) for CBD 25mg/kg/day vs. placebo. 28.4% (p=0.002) for CBD 50mg/kg/day vs. placebo	CBD 25 or 50mg/kg/day for 14 weeks	Thiele et al.^21^

CBD: cannabidiol; DS: Dravet syndrome; LGS: Lennox-Gastaut syndrome; TSC: tuberous sclerosis.

The use of CBD has limitations and needs further studies to elucidate its use as routine therapy in the treatment of epilepsy and seizures. Although studies suggest efficacy for a wide variety of types of seizures and types of epilepsy,^22–26^ to date, robust evidence of clinical benefit is limited to seizures in patients with DS and LGS and for patients with seizures associated with TSC. Therefore, the use for other seizure types, such as focal epilepsy and idiopathic generalized epilepsy, which are the most common, should be considered an investigation. In all controlled studies performed to date, CBD has been administered as adjuvant therapy in patients with persistent seizures undergoing treatment with other anticonvulsants. Due to this fact, additional studies of CBD as a drug in monotherapy and studies including direct comparisons with alternative treatment options are necessary, given the remarkable emergence of other drugs for the same therapeutic indication. In the pediatric population, future studies are needed to demonstrate the potential consequences of children’s exposure to CBD in the early life period. Another issue is that studies that used CBD together with clobazam showed a significant increase in norclobazam, the active metabolite of this benzodiazepine, and it is necessary to determine in future studies the contribution of CBD and norclobazam in the control of seizures.^22^

One of the biggest concerns regarding the use of CBD is its adverse effects in the short and long terms. Such effects are difficult to assess, and the scientific evidence generated so far is not robust and has several biases. The absence of controlled studies and the frequent use of other antiepileptic drugs by CBD users make it difficult to interpret the data since they have their intrinsic side effect profile and have several drug interactions with CBD. In this scenario, studies show that adverse effects are common in patients using CBD and occur with high frequency in patients using placebo, possibly due to the polypharmacy and severity of the disease of the studied patients.^27^

In one of the most important studies regarding the use of CBD in the pediatric population, Devinsky et al.^17^ demonstrated in a clinical trial, in line with the other data in the literature, a satisfactory safety and tolerance profile to the drug, with only 3% discontinuation of use for side effects. Adverse effects were reported in 79% (128/162) of the patients, with the vast majority being mild/moderate and transient, demonstrating a profile favorable to the use of CBD. The most common adverse effects were drowsiness (41%), decreased appetite (19%), diarrhea (19%), and fatigue (13%). Serious adverse effects were occurred in 30% of patients, including status epilepticus (6%), severe diarrhea (2%), and significant weight loss (1%). Table 2 shows the main effects of CBD use described in the literature.

**Table 2:** Main adverse effects of cannabidiol use described in the literature.

	Devinsky et al.^17^	Devinsky et al.^18^	Thiele et al.^19^	Devinsky et al.^20^		Thiele et al.^21^	
CBD oral dose (mg/kg/day)	20–50	20	20	10	20	25	50
Number of patients	162	61	86	67	82	75	73
Somnolence, n (%)	41 (25)	22 (36)	12 (14)	14 (21)	25 (30)	10 (13)	19 (26)
Decreased appetite, n (%)	31 (19)	17 (28)	8 (9)	11 (16)	21 (26)	23 (31)	41 (56)
Diarrhea, n (%)	31 (19)	7 (11)	11 (13)	7 (10)	12 (15)	15 (20)	17 (23)
Upper respiratory tract infection, n (%)	–	7 (11)	–	11 (16)	11 (13)	7 (9)	7 (10)
Pyrexia, n (%)	–	9 (15)	–	6 (9)	10 (12)	14 (19)	12 (16)
Vomiting, n (%)	–	9 (15)	6 (7)	4 (6)	10 (12)	13 (17)	13 (18)
Status epilepticus, n (%)	13(8)	–	–	7 (10)	4 (5)	–	–
Convulsion, n (%)	18 (11)	7 (11)	–	–	–	5 (7)	8 (11)

CBD: cannabidiol.

To that end, in a systematic review with meta-analysis, Reis et al.^28^ demonstrated that the most frequent adverse effects were diarrhea, drowsiness, and decreased appetite, being more frequent at the beginning of treatment, in line with the systematic review published by Alexis Arzimanoglou et al.^14^ in 2020, which demonstrated that adverse effects were more common in the first 2 weeks of drug use.^28^ Likewise, side effects appear to be greater in patients using higher doses of CBD.^29^

The interaction between CBD and several hepatic metabolic pathways is also a matter of great debate and may be responsible for some of the side effects observed in the series. CBD inhibits the CYP2C19 enzyme, responsible for metabolizing *N*-desmethylclobazam, an active metabolite of clobazam, thereby increasing its plasma concentration. It is suggested in the literature that this interaction is responsible for the high incidence of drowsiness in patients who use both drugs concomitantly.^28^

In addition, CBD, being metabolized by CYP450, acts as an inhibitor of this enzyme, decreasing the serum levels of carbamazepine and phenytoin and increasing those of valproate, clobazam, ethosuximide, topiramate, rufinamide, zonisamide, perampanel, and eslicarbazepine. However, the clinical significance of these interactions is still uncertain and does not appear to be harmful.^14^

In a study conducted by Gaston et al.^30^ in 2019, the researchers prospectively evaluated 132 patients with refractory epilepsy. Participants were divided into groups, in which patients were evaluated using CBD with or without clobazam and CBD with or without four other antiepileptic drugs: rufinamide, eslicarbazepine, zonisamide, and topiramate. The authors concluded that there was no difference in controlling the frequency or severity of epileptic seizures in different groups. This finding reinforces the hypothesis postulated in the literature that the interaction between CBD and other drugs is not of great clinical relevance.

Given this, as scientific evidence is scarce, patients using CBD should be systematically evaluated by their doctors, assessing treatment tolerance, adherence, and effectiveness in the context of drug interaction.^14^

Still, concerning liver function, patients using CBD may have elevated transaminases. In most cases, the increases are self-limiting and do not lead to discontinuation of treatment. Most of these patients (79–100%) make concomitant use of valproic acid, raising the hypothesis that CBD potentiates liver damage caused by this drug.^14,27^ The elevation usually occurs in the first 2 months of CBD use, being resolved with CBD discontinuation and/or valproate in most cases.^29^ Such data reinforce the hypothesis that many of the side effects reported using CBD are caused or exacerbated by the concomitant use of other antiepileptic drugs.

It is unclear whether adverse neurological effects, such as cognitive deficits, decreased volition, and the development of psychiatric illnesses, occur only in patients using another endocannabinoid-tetrahydrocannabinol (THC) — or whether long-term use of CBD may also be associated with these effects.^1^ Notably, 96-week follow-up data show good tolerance and a favorable profile of side effects to CBD use, but studies are lacking to assess the long-term effects, especially in the pediatric population.^31^

Although the incidence of side effects reported in the studies is high, most studies indicate a good safety profile and tolerance to the drug, with most adverse effects being mild/moderate and transient. The limitations of the review are the use of studies with different methodologies and designs, divergent doses of CBD, small sample size, and few assessments of adverse effects, which makes the comparison between them difficult. Therefore, to ratify the use of CBD and increase safety in the prescription of this drug, studies with larger samples and long-term follow-ups are necessary.

The treatment of refractory epilepsies represents a significant challenge, showing the importance of developing new therapeutic options. Since the ECS seems to be involved in the pathophysiology of epilepsy, CBD is promising since it has presented satisfactory results in crisis control in well-designed RCT studies. However, there are main concerns with the use of CBD as a therapy for children with epilepsy, such as the unknown long-term adverse effects, critical drug-drug interactions (especially clobazam), and lack of regulatory oversight of retail unlabeled CBD-enriched products. The use of non-GMP/GLP highly purified compounds, with erratic CBD-enriched cannabis extract oils, may produce other phytocannabinoid molecules (such as THC) with harmful long-term potential. All doses described in this article are recommended by the manufacturer or are off-label doses used in clinical trials, and it is not a recommendation for use. Therefore, further clinical long-term, placebo-controlled trials using pharmaceutical-grade CBD are necessary and opportune to prove the effectiveness, safety, and absence of severe long-term adverse effects in pediatric populations.
